# The role and need for psychological support in the treatment of adolescents and young people suffering from type 1 diabetes

**DOI:** 10.3389/fpsyg.2022.945042

**Published:** 2023-01-04

**Authors:** Magdalena Małachowska, Zuzanna Gosławska, Ewa Rusak, Przemysława Jarosz-Chobot

**Affiliations:** ^1^Students' Scientific Association at the Department of Children's Diabetology, Medical University of Silesia, Katowice, Poland; ^2^Faculty of Medicine, The Medical University of Warsaw, Warsaw, Poland; ^3^Department of Clinical Endocrinology, Independent Public Health Care Central Clinical Hospital of the Medical University, Łódź, Poland; ^4^Department of Children's Diabetology, Medical University of Silesia, Katowice, Poland

**Keywords:** diabetes type 1, psychology, support, adolescents, management, care

## Abstract

Psychological support might be perceived as one of the most important factors in the treatment of people suffering from type 1 diabetes, particularly among vulnerable groups such as adolescents and young people. Problems arising from extreme pressure put on young patients, high expectations, and specific limitations associated with diabetes often reflect in negative wellbeing and affect patients' behavior, resulting in lower self-esteem, mood swings, depression, or even eating disorders. Therefore, the need for a more holistic approach to the treatment of diabetes and caring about psychological support can be observed, which may contribute to better functioning and management of the disease. Differentiation of certain approach methods such as the positive approach (PA) discussed in the text may help young patients in motivation and coping with their disease as well as accepting limitations caused by type 1 diabetes. This would decrease the risk of potential revolt against medical recommendations, common for patients at the mentioned age, and help raise awareness of the problem. Maintaining life balance through undertaking regular physical activities and being open to new strategies such as telenursing can also result in the improvement of glycemic control. The studies presented have proven the great effectiveness of personalized care adjusted to the patient with psychological support, as well as the invaluable role of education in diabetes, which includes not only standard procedures such as calculating an appropriate insulin dose but also the invention of effective coping mechanisms, which influence patients' performance and wellbeing.

## Introduction

Treatment of chronic disease is an extremely challenging and demanding process that not only requires a holistic approach but also fully engages the patients and their relatives who contend with constant pressure and the need for responsibility. The situation becomes even more complicated when it comes to younger patients who, very often, function with their disease from an early age and are forced to accept the limitations connected with diabetes and adapt to them with the passing of time. As type 1 diabetes is said to be one of the most frequent chronic diseases affecting children and adolescents nowadays, it causes a lot of problems that young people must face on an everyday basis (Anderzén et al., 2020). Because they are sometimes not able to deal with the mentioned situation on their own and feel overwhelmed, they may require some additional help to enable them to function better.

The aim of this publication is to provide certain examples presenting the problems of adolescents suffering from type 1 diabetes, as well as the mechanisms of their behavior, which would help in assessing the need for potential psychological aid. Also, specific psychological approaches and solutions will be presented to observe the impact of psychological support on patients' attitudes toward treatments and their wellbeing, which are very important in achieving success in therapy.

## Need for psychological support

Certainly, adolescence can be perceived as a remarkably complicated period in the life of a person with diabetes. Apart from both physical and psychological changes, they may also encounter new limitations arising from their disease that they were not aware of before. These may have an impact on functioning, wellbeing, and the way in which they perceive their disease. In addition, for many adolescents, the constant pressure may often result in an increased level of stress and a decrease in the level of quality of life. As a consequence, it is highly probable that adolescents become disobedient, which may result in worsening their self-control and not adhering to recommendations. Baucom et al. (2015) reported that ~60% of adolescents suffering from type 1 diabetes present mentioned behavior as they are not fully engaged in the process of their treatment and do not strive to achieve their blood glucose level values within an advisable range. Moreover, another study performed in the United States showed that among young people with diabetes aged between 13 years and 18 years, only 20% maintain glycemic control on an appropriate level proposed by American Diabetes Association (Rose et al., 2020). Although these data differ depending on the country, health center, and the level of medical care that they offer, the general trend in adolescents' course of action is similar. Considering this fact, it is necessary to draw attention to the role of psychological support in the treatment of patients.

## Impact of psychological support on diabetes management

Management of diabetes is often mostly associated with insulin infusions, usage of specific electronic devices, and measuring blood glucose levels. Nonetheless, the treatment of diabetes should be considered holistically with psychological support, playing an important role in the treatment. As Henríquez-Tejo and Cartes-Velásquez (2018) state, psychological support can be treated as part of the treatment of type 1 diabetes. It may not only help in managing the condition but also in reducing the risk of diabetes complications, which is an important long-term aim in the treatment (Dimitri et al., 2020). Henríquez-Tejo and Cartes-Velásquez (2018) also mention the positive impact of therapies and visits to psychologists on the willingness to stick to medical recommendations, which shows the importance of using certain coping mechanisms and search for psychological strategies that might help in wellbeing and diabetes management. Positive affect intervention is an example of a strategy that provides patients with more advanced psychological care than the standard one. It is based on developing a positive attitude toward treatment as well as improving self-esteem and practicing gratitude exercises that involve not only adolescents but also their parents (Jaser et al., 2019). At first, certain questionaries are filled by patients with questions such as *What is something that you are proud of?*, which aim for specifying the topics for positive approach exercises and preferable means of practicing specific skills connected with gratitude, self-affirmation, and confidence (Jaser et al., 2019). Apart from exercises, parents are asked to participate in the therapy by building the child's confidence through regular appreciation of small successes not only connected with the child's management of diabetes but also everyday functioning. Patients are advised to focus on their achievements and avoid feeling guilty when not reaching their goals. The study showed that such an approach contributes not only to the improved quality of life of patients but also to greater motivation and engagement in the treatment. Moreover, it was demonstrated that the increase in the frequency of blood glucose monitoring by one per day is two times more possible in the case of patients with positive affect intervention applied than those that were not provided with any additional psychological support (Jaser et al., 2019). It suggests that this strategy might have an impact on the frequency of blood glucose monitoring in the longer term; however, this would require additional modifications to the method as well as more intensified interventions.

Another study investigating the relationship between adolescents' attitudes toward diabetes and their treatment effects has indicated some correlations. It has been observed that higher self-esteem, self-efficacy, and sense of satisfaction, caused by successful compliance with diet, often result in greater and more upright further adherence to medical recommendations (Nouwen et al., 2009). It reflects in the less frequent occurrence of diabetes complications, comorbidities, sense of restlessness, and increased willingness to keep the diet (Nouwen et al., 2009). This favors the belief that an individual approach to the patients, working on their confidence, an appropriate way of communication, and motivation can positively influence metabolic control and the life quality of people with diabetes.

The importance of direct support is also presented by Kotsani et al. (2018) who performed a study whose aim was to analyze the impact of telenursing on the frequency of blood glucose measurements and improvement in blood glucose variation. In the mentioned study, young people suffering from type 1 diabetes aged 18–39 years were called once a week by diabetes specialist nurses. During these calls, patients could ask for potential help and talk about their problems connected with diabetes management. They were also given personalized guidelines based on their glucose values that were shared with the nurses. It was observed that, after 3 months of mentioned phone calls, patients presented increased obedience toward medical recommendations and adherence to self-monitoring (Kotsani et al., 2018). There was no similar pattern observed in the controlled group of patients, which again proves that different forms of support—in this case, direct talk with a diabetes specialist and a personalized approach tend to influence positively the treatment and patients' performance (Kotsani et al., 2018). Moreover, the presented study indicated the principal role of specialized diabetes nurses in the support of patients as well as appropriate diabetes education, which together form a basis for the appropriate management of the disease. It is not only crucial in the development of appropriate habits at the beginning of diabetes but also in maintaining them in adulthood.

## Challenges facing adolescents and young patients

Technological tools and devices that are widely used by patients with diabetes nowadays help them in normal functioning and making appropriate treatment decisions. They make it easier for patients to alleviate the impact of certain factors on blood glucose levels, such as the miscalculation of the amount of carbohydrate intake or undergoing sudden physical exertion. However, there are still factors that significantly affect blood glucose levels and are out of the patient's control, such as emotions. Helgeson et al. (2009) highlighted the impact of relations with peers on adolescents' attitudes toward glycemic control and self-discipline. They emphasized the interaction between psychological wellbeing and blood glucose control, which in many cases are negatively influenced by conflicts with friends. This study also presented girls to be more prone to such associations. Moreover, apart from relations with peers, which are extremely important for adolescents, family conflicts related to type 1 diabetes contribute to higher HbA1c values in some cases, which prove that they should be taken into consideration in the treatment process (Lansing et al., 2018). Therefore, it is reasonable to state that placing emphasis on permanent psychological support and its normalizing in all health centers could help in developing specific mechanisms and rituals that could be used to successfully cope with stressful situations, when needed, and improve patients' wellbeing.

Adolescents are more vulnerable to psychiatric disorders due to the challenges, potential difficulties in functioning, and changes that they must face. In addition, dealing with chronic diseases such as type 1 diabetes makes the patients even more sensitive, as Buchberger et al. (2016) found that they are two times more susceptible to suffer from anxiety, depression, and other disorders of this type. Living under the constant supervision of physicians and parents creates pressure for young patients with diabetes who feel the need to meet the demands and adhere to medical recommendations. Because of this fact, at some point, they may feel overwhelmed because, in comparison to their peers, they have more responsibilities and limitations, which may cause them to feel unfair. Sometimes, adolescents feel excluded from the group due to their need for performing specific lifestyles, planning meals, and maintaining steady control, which does not apply to their peers. As a consequence, in certain situations, the disease started to be perceived as a burden, which results in the occurrence of mood swings and the development of possible mental disorders, which concern a significant number of young patients with diabetes. Bernstein et al. (2013) present a study in which 35% of 150 adolescents admit to having experienced one of the mentioned mental disorders, including depression, anxiety, and eating disorders. Moreover, 15% of the reports have at least two of them, which proves the seriousness of the situation (Bernstein et al., 2013). Finally, ~25% of those tested had a moderate or high indicator of the Center for Epidemiologic Studies Depression Scale (Bernstein et al., 2013). The negative influence of depression on compliance with medical recommendations was also stated (Bernstein et al., 2013); therefore, mental disorders must be treated and diagnosed to improve patient's results and avoid any complications. De Ornelas Maia et al. (2012) highlight the need for the investigation of psychiatric disorders in patients with diabetes while also indicating the differences in the nature of problems that patients with diabetes deal with depending on the type of diabetes. The mentioned pressure from constant control and high expectations of physicians and parents can be problematic, but at the same time, it also motivates young patients, helps them in adherence to recommendations, and improves their self-discipline. It is beyond doubt that more profound research would enable physicians and medical workers to investigate more effective and defined methods of help to counteract and prevent psychiatric diseases among patients in the future.

## Key role of psychological care in preparation for healthcare transition

There is no doubt that adolescence is a very demanding and complicated time in the lives of young patients and the management of their diabetes. However, attention should be also paid to the process of transition between pediatric and adult healthcare. According to Henríquez-Tejo and Cartes-Velásquez (2018), the switch in the type of care very often results in the deterioration of diabetes control, which is presented by decreased motivation as well as worsening of self-control and resilience, which all result in decreased life quality. Such an approach is considered to be caused by changes in patients' personal lives connected to lifestyle, communities, and environment the patients live, unstable working conditions, and future perspectives (Kotsani et al., 2018). This proves how different life circumstances impact mental health, which as a consequence can be reflected in the way in which patients manage their disease (Kotsani et al., 2018). Similar conclusions are presented by Sheehan et al. (2015) who report the change in patients' attitudes toward the treatment as they become adults. This was observed in the study performed on 229 young adults whose rate of attendance decreased by 49% 2 years after the transition between pediatric and adult healthcare (Sheehan et al., 2015). Moreover, some studies indicated significant gaps in the attendance to appointments of young adults with one study reporting 34% of 258 young people to have missed their visits for 6 months and 13% having a gap of not less than a year (Garvey et al., 2013). The presented trend in the behavior of young patients with diabetes is bothering and suggests the potential need for psychological support at the very stressful time of transition into adult healthcare. Young adults should be prepared for the challenges arising from the transition and be aware of possible complications to make easier adaptation to the new situation, which, as a consequence, would help them to feel safe and maintain a stable level and quality of treatment. It should be emphasized that psychological support might be also important to young patients who did not receive enough help and assistance from their families and relatives when living with diabetes as children. As a consequence, very often, they might be miseducated and have poor glycemic control. In such cases, implementing psychological support during childhood might help in coping with diabetes, understanding the need for self-care and becoming responsible for their treatment when they become adults, which could surprisingly improve patients' performance. Unfortunately, though the need for psychological care in relation to healthcare transition is understandable, there are still not many protocols and general recommendations, which might facilitate implementing such strategies (Henríquez-Tejo and Cartes-Velásquez, 2018). According to the Pediatric Society of Chile, special teams consisting of specialists who would be responsible for controlling diabetes management of young adult patients as well as educating them and encouraging them to maintain control of their diabetes should be created to make healthcare centers more effective (Henríquez-Tejo and Cartes-Velásquez, 2018).

## Self-perception and body image in type 1 diabetes

Critically, a significant number of people suffering from a chronic disease, including type 1 diabetes, struggle with low self-esteem and inferiority complexes connected with their weight and appearance (Lašaite et al., 2016). Visible sites of infusion sets, injections, and sensors may cause negative emotions. Awareness of being dependent on specific equipment and accessories that require constant engagement and self-control sometimes becomes irritating and overwhelming. It has been observed that insulin pumps bring varied emotions depending on the level of glycemic control of the patients (Ritholz et al., 2007). For some patients, the insulin pump was accompanied by positive emotions as it helped them in maintaining their glucose levels more stable as well as normalizing relations with food and feeling a sense of freedom in functioning (Ritholz et al., 2007). In the same study, a group of participants admitted that, due to the insulin pump, they felt more comfortable discussing their disease and were not feeling ashamed of it. In contrast, those with higher HbA1c expressed being “tired” of the machine and presented intentions of removing it. Also, the study showed that some first contact with an insulin pump brought feelings of the onset of the disease and negative emotions that accompanied the patients at the time when they were diagnosed with diabetes. Mainly, women presented worries connected with the impact of insulin pump on their self-acceptance and body perception. They aim for hiding the machine and admitted to having difficulties in accepting it as they perceive the machine as an obstacle and “fashion challenge” (Ritholz et al., 2007). It proves that adapting to living with the disease and its limitations is an ongoing and demanding process and should be controlled (Ritholz et al., 2007). Though the study was presented on adults, it demonstrates the type of problems and emotions that children and adolescents might face in the future, which points to the need to provide psychological support at a young age, as it may facilitate better functioning with diabetes in adult life.

In fact, one of the most important aspects of the treatment of diabetes is adherence to recommended diet and paying attention to the food composition and its quality. Very often, the pressure connected with appropriate nutritional values and calories calculation has a negative impact on the attitude toward food and eating, which results in the development of eating disorders. Although a majority of cases involve girls and young women, men can also be affected by eating disorders and, as with mental disorders, those suffering from diabetes are two times more vulnerable to experience eating-related disorders than their peers (Markowitz et al., 2010). In any event, such an eating disorder must be considered a serious problem, mainly due to the strategies that adolescents decide to apply. These involve the intentional omission of insulin doses to lose weight, binge eating, which results in significant fluctuations in blood glucose levels, bulimia, and undergoing excessive physical effort. As they all have a negative impact on the functioning of patients and their wellbeing, it is necessary for medical workers, parents, and adolescents themselves to be able to observe alarming symptoms and react to them as soon as possible. However, as Markowitz et al. (2010) notice, it makes it harder to diagnose people with diabetes and eating disorders because partial dietary restrictions, weight control, calculating nutritional values, and other actions that may indicate the onset of an eating disorder are considered normal when it comes to the management of type 1 diabetes. It also requires more effort to cure eating disorders in a patient with diabetes as common strategies such as resigning from the calculation of nutritional values and calories cannot be applied for them because it is necessary for their treatment. Given the fact that appearance and body shape play a very important role in adolescents' lives nowadays, it is highly expectable that the need for psychological support in this field will increase in future. Young people will be more vulnerable to criticism; therefore, the incidents of eating disorders and other problems connected with lowered self-esteem may occur more frequently. That is why it is necessary to educate young people and their parents so they could develop specific strategies that will help them to deal with such problems and prevent them in future.

## The role of regular physical activity in dealing with diabetes

Successful long-term management of diabetes requires a holistic approach with regular physical activity being a part of its components. According to Czenczek-Lewandowska et al. (2018), regular physical exercise not only reduces the risk of secondary comorbidities but also influences maintaining blood glucose levels within range and promotes general wellbeing. At the same time, the study performed by Montt-Blanchard et al. (2022) alarms about a low percentage of patients, accounting for 17.8% who are physically active and ~60% of patients who do not even achieve the minimum recommended time of physical activity. One of the barriers that discourage patients to perform activities includes fear of hypoglycemia, lack of time, motivation, and knowledge about dealing with diabetes on exertion Montt-Blanchard et al. (2022). This proves that appropriate education is extremely important in the process of diabetes management and should be a part of treatment from an early time of the onset of the disease, which unfortunately depends on center readiness and the competences of medical staff (Dash et al., 2020). If a person would be appropriately acquainted with procedures that should be followed by hypoglycemia, they might be more confident and be willing to undertake physical activity more often. It is also presented that through performing physical exertion, people suffering from diabetes create their own strategies, which aim for maintaining blood glucose levels within range, based on the experience and the data obtained during past workouts (Montt-Blanchard et al., 2022). It could be compared with a closed loop as people develop strategies that result in appropriate blood glucose management during exertion, they become more motivated to exercise in the future. As a result, their confidence and self-efficacy increases as well as improves their wellbeing. Therefore, physical activity contributes to improving resilience, which is extremely important in everyday life functioning when suffering from type 1 diabetes. The statement of one of the participants of the study “diabetes started bleeding into my identity through running (…) [diabetes] is making me utilize my creativity as a primary self-identifier” suggests that, for some people, running becomes a means of coping with diabetes and helps in the acceptance of the disease (Montt-Blanchard et al., 2022, p. 7). It can also play a role in shaping a patient's character and motivation, which is reflected in improved wellbeing and mental health. It must be noted that, though the only discipline considered in the study was running, other forms of physical activity are expected to also positively influence patients' performance and wellbeing. Referring to combat sports, Witkowski et al. (2016) mentioned the significant role of the coach in the development of personality traits and its influence on such aspects of health as somatic, mental, and social health, which again indicates the link between the performance of the physical activity and mental health. This also proves that psychological support does not need to be provided only by medical staff, but the role of other people often considered as patients' role models is very important as well. These people can help and assist patients in everyday life situations, which make them invaluable supporters.

## Discussion

Type 1 diabetes is a very challenging disease that engages both patients and their relatives to a greater extent. Although it is not visible at first, despite the presence of devices such as insulin pumps or sensors, it is a serious disease that often affects patients' lives, their functioning, and priorities as well as some choices and decisions. Because of common early onset, diabetes has a significant role in further functioning in society or even creating interpersonal relationships (Cousino et al., 2013). It requires engagement from the first day of diagnosis and puts a huge responsibility on patients and their parents, which makes the time of the onset of the disease even more stressful and demanding. As it was presented, due to the nature of the disease, patients with diabetes are more likely to experience some psychological disorders as they face much more challenges, stress, and pressure than other people, especially at the specific age of adolescence. Given the examples, it could be observed that additional psychological care helps the patients to deal with their problems and function as healthy ones. The improvement in glycemic control and upright adherence to medical recommendations following the application of an individual approach to the patient and psychological care prove that psychological support in adolescents with type 1 diabetes plays a significant role in the process of treatment and influences their attitudes toward disease in a positive way. Certainly, psychological care should form an integral part of the process of treatment as all studies presented have shown its positive impact on wellbeing of adolescent patients and their performance. Although the awareness and knowledge about the role of mental health in the treatment of other diseases increases, it is still often not treated seriously enough. Meanwhile presented correlations showed that effective medical healthcare can only be achieved through the application of a holistic approach and providing patients with psychological support. Being aware of potential problems that adolescents with diabetes deal with might not only help in the early detection of certain psychiatric disorders but also may enable them to appropriately react to them to alleviate their impacts on patients' wellbeing and glycemic control. Understanding the need for psychological support in children suffering from diabetes might be also important for the ones that are resistant to obtaining psychological support and applying innovative methods that are to help in diabetes management. Though the results of presented studies are in favor of the positive role of psychological support and holistic approach in the treatment, it must be noted that some patients and their caregivers will be skeptical and not feel convinced to incorporate the given approach, which is why they should be constantly educated and their awareness about the role of psychological support should be raised. It could be also done by helping them realize that people suffering from diabetes do not need to be diagnosed with specific psychiatric conditions and psychological support should be normalized and treated rather as something natural (Gonzalez et al., 2016).

## Conclusion

Given the above findings, it can be stated that psychological support plays an important role in the treatment of young people suffering from type 1 diabetes. Not only does it influence patients' wellbeing but also improve attitudes toward the treatment and reflects in better adherence to medical recommendations. Certainly, the arguments presented earlier prove that psychological care should be treated as an integral part of the treatment of diabetes and should not be neglected when it comes to its impact on a patient's condition and management of the disease. Therefore, the need for a holistic and individual approach should be emphasized more at medical centers as it positively influences glycemic control and often results in better functioning of patients. There is no doubt that not all health centers have conditions that enable medical staff to convey enough knowledge about diabetes to patients in a short period of time and manage to provide them with personalized medical care and a holistic approach. As these are considered to be the key components in the treatment success of diabetes, all health centers should aim for improving the quality of medical care that they offer together with the formation of professional medical teams consisting of diabetologists, endocrinologists, educators, dieticians, and psychologists.

## Author contributions

MM, PJ-C, and ZG: article conception and design, bibliography collection, and manuscript preparation. ER: article conception and design, bibliography collection, manuscript preparation, and supervision. All authors contributed to the article and approved the submitted version.
